# MicroRNAs as Important Players and Biomarkers in Oral Carcinogenesis

**DOI:** 10.1155/2015/186904

**Published:** 2015-10-04

**Authors:** Anjie Min, Chao Zhu, Shuping Peng, Saroj Rajthala, Daniela Elena Costea, Dipak Sapkota

**Affiliations:** ^1^Department of Oral and Maxillofacial Surgery, Xiangya Hospital, Central South University, Changsha, China; ^2^School of Stomatology, Central South University, Changsha, China; ^3^Cancer Research Institute, Central South University, Changsha, China; ^4^The Gade Laboratory for Pathology, Department of Clinical Medicine, Faculty of Medicine and Dentistry, University of Bergen, 5021 Bergen, Norway; ^5^Centre for Cancer Biomarkers (CCBIO), Faculty of Medicine and Dentistry, University of Bergen, 5021 Bergen, Norway; ^6^Department of Pathology, Haukeland University Hospital, 5021 Bergen, Norway; ^7^Department of Oncology and Medical Physics, Haukeland University Hospital, 5021 Bergen, Norway

## Abstract

Oral cancer, represented mainly by oral squamous cell carcinoma (OSCC), is the eighth most common type of human cancer worldwide. The number of new OSCC cases is increasing worldwide, especially in the low-income countries, and the prognosis remains poor in spite of recent advances in the diagnostic and therapeutic modalities. MicroRNAs (miRNAs), 18–25 nucleotides long noncoding RNA molecules, have recently gained significant attention as potential regulators and biomarkers for carcinogenesis. Recent data show that several miRNAs are deregulated in OSCC, and they have either a tumor suppressive or an oncogenic role in oral carcinogenesis. This review summarizes current knowledge on the role of miRNAs as tumor promotors or tumor suppressors in OSCC development and discusses their potential value as diagnostic and prognostic markers in OSCC.

## 1. Introduction

Head and neck squamous cell carcinoma (HNSCC) consists of a heterogeneous group of malignancies arising from oral cavity, nasal cavity, paranasal sinuses, pharynx, larynx, and salivary glands. Oral cancer, represented mainly by oral squamous cell carcinoma (OSCC), is the most common type of HNSCC. OSCC is the eighth most common cancer worldwide accounting for more than 300,000 new cases and 145,000 deaths in 2012 [1]. Usually, OSCC detection depends on the clinical examination of oral cavity, followed by a biopsy for histological analysis. However, despite the easy access for visual examination, OSCC is often detected at advanced stages leading to severely reduced patient survival. In spite of the recent advances in diagnosis and treatment modalities, less than 50% of OSCC patients survive for 5 years [2]. Late diagnosis, regional lymph node metastasis, and recurrences are the major causes related to the poor prognosis and reduced survival for OSCC patients [3, 4]. Thus, reliable molecular markers that can (i) provide earlier and more precise OSCC diagnosis, (ii) predict prognosis, and (ii) assign patients to the best-targeted treatment available are urgently needed.

**Figure 1:**
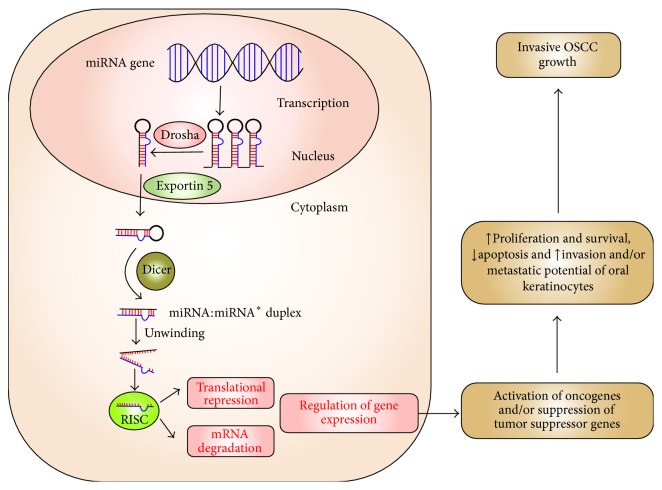
Schematic illustration demonstrating biogenesis and function of miRNA. miRNA genes are transcribed into primary miRNA (pri-miRNA) by RNA polymerase III. These miRNAs are further converted into second precursors (pre-miRNA) by Drosha and are exported into cytoplasm by Exportin 5. Additional processing by Dicer produces miRNA:miRNA^*∗*^ duplex. Only one strand of miRNA:miRNA^*∗*^ duplex is preferentially assembled into the RNA-induced silencing complex (RISC). RISC acts on target mRNA(s) and leads to either translational repression or mRNA cleavage. Suppression of tumor suppressive genes and/or activation of oncogenes by miRNA lead to excessive cell proliferation and survival, increased antiapoptosis, and enhanced invasive and metastatic potential of oral keratinocytes, resulting into invasive cancerous growth.

For almost three to four decades, changes in protein coding tumor suppressor genes and/or oncogenes have been thought to be the main drivers of tumor development [5, 6]. However, the recent discovery of thousands of genes that transcribe noncoding RNAs (including miRNAs) makes it obvious that cancer biology is even more complex than initially expected. Several layers of molecular regulators (e.g., mRNA, miRNA, and protein) are involved in the development and maintenance of cancerous phenotypes. Among them, miRNAs, 18–25 nucleotides long, noncoding RNA molecules [7–9], have recently gained significant attention as potential regulators and biomarkers for human carcinogenesis. At the molecular level, miRNA binds to 3′-untranslated region (3′-UTR) of target mRNA(s) and suppresses its expression by either translational repression or mRNA cleavage [10] (Figure 1). A single miRNA can regulate expression and/or function of hundreds of target mRNAs and proteins and regulates several biological processes (e.g., cell proliferation, differentiation, migration, apoptosis, and signal transduction) important for cancer development [8, 11–13] (Figure 1).

Many recent studies have shown deregulated expression of miRNAs in OSCC and OSCC-derived cell-lines compared to their normal counterparts, indicating their potential role in oral cancer development. Accordingly, several miRNAs have been shown to function either as tumor suppressors or as tumor promoters in OSCCs (reviewed in [14, 15]). In addition to their key biological functions in OSCC tumorigenesis, expression levels of several of miRNAs have been shown to correlate with clinicopathological variables [16] and to have a diagnostic and prognostic value in OSCC [15, 17]. For these reasons, miRNA has been a hot topic in cancer research for the last few years and several studies about miRNAs in OSCC have been published recently, as summarized in Table 1. The current review aims to highlight the oncogenic and tumor suppressive roles of miRNAs in OSCC development and discusses their potential value as diagnostic and prognostic markers for OSCC management.

## 2. Methods

Literature search was performed by using the PubMed database. Following key words were used for the literature search: “oral cancer and miRNA,” “oral cancer and microRNA,” “oral squamous cell carcinoma and miRNA,” and “oral squamous cell carcinoma and microRNA.” Exclusion criteria were articles not related to OSCC/HNSCC and/or miRNA, purely descriptive articles, articles lacking clinical pathological correlation, and/or articles for which full texts were not available in English. Only clinically relevant articles published within April 2015 were included in this review. Additionally, individual articles retrieved manually from the reference list of the relevant papers were also included.

## 3. miRNAs as Oncogenes in OSCC

A number of miRNAs have been shown to be upregulated in OSCC and to function as oncogenes. A well-studied miRNA, the miR-21, has been shown to be overexpressed and to regulate several biological functions in OSCC [16, 18–20]. Overexpression of miR-21 has also been observed in oral premalignant lesions (oral leukoplakia) compared to normal oral mucosa, indicating that alteration in miR-21 could be an earlier event in OSCC progression [21]. A number of* in vitro* and* in vivo* experimental data have demonstrated an oncogenic role of miR-21 in OSCC by promoting cell proliferation [22], invasion [18, 23], antiapoptosis [16], and chemoresistance [24]. These oncogenic functions were shown to be regulated by miR-21-mediated downregulation of several established tumor suppressor molecules, including PTEN [25], programmed cell death 4 (PDCD4) [23], tropomyosin [16], reversion-inducing cysteine-rich protein with kazal motifs (RECK) [26], and dickkopf 2 (DKK2) [18]. In addition to the functional roles in OSCC cells, a growing body of evidence suggests that miR-21 might be important in the regulation of carcinoma associated fibroblasts (CAFs) induction and their activity [20, 27]. miR-21 was shown to be predominately localized in OSCC stroma and colocalized with *α*-smooth muscle actin positive CAFs. Additionally, higher stromal expression of miR-21 was associated with poor prognosis in OSCC [20].

miR-31 and its passenger strand miRNA (miR-31^*∗*^) have been shown to be upregulated in oral leukoplakia (OLP) and OSCC and to have an oncogenic role in OSCC tumorigenesis [28–31]. Liu et al. demonstrated that ectopic expression of miR-31 repressed its target factor-inhibiting hypoxia-inducible factor (FIH) expression to activate hypoxia-inducible factor (HIF) under normoxic conditions, both* in vitro* and* in vivo*. Additionally, miR-31-FIH-HIF-VEGF regulatory cascade was found to affect several biological processes such as cell proliferation, migration, and epithelial-mesenchymal transition (EMT) in OSCC cells [28]. Moreover, miR-31 was shown to collaborate with human telomerase reverse transcriptase (hTERT) to immortalize normal oral keratinocytes (NOKs), indicating that it might contribute to early stage oral carcinogenesis [31]. Similarly, miR-31^*∗*^ regulated apoptosis, cell proliferation, migration, and invasion in OSCC cells [29]. These miR-31^*∗*^ regulated functional effects were mediated by the regulation of fibroblast growth factor 3 (FGF3) [29] and RhoA [32] expression levels.

miR-146a has been demonstrated to be overexpressed in OSCC and to enhance OSCC tumorigenesis both in the* in vitro* and* in vivo* mouse xenograft model [33, 34]. The oncogenic functions of miR-146a were found to be associated with concomitant downregulation of IL-1 receptor-associated kinase 1 (IRAK1), TNF receptor-associated factor 6 (TRAF6), and NUMB [33]. A previous study from the same group suggested an association between a higher OSCC miR-146a expression and nodal involvement in patients carrying C polymorphism (rs2910164) [34]. However, findings from Palmieri et al. indicated that the rs2910164 polymorphism is not associated with OSCC progression [35]. Further investigations are needed to clarify a possible role of the variant allele or rs2910164 in OSCC progression.

miR-134 expression was upregulated in HNSCC tissue specimens and cells (HSC-3, OECM-1, and SAS cell-lines) compared to the corresponding normal controls. Functional analysis revealed that miR-134 expression enhanced the oncogenicity of HNSCC cells* in vitro* as well as tumor growth and metastasis of HNSCC cells* in vivo* via targeting WW domain-containing oxidoreductase (WWOX) [36]. In another study, miR-155 was found to be overexpressed in OSCC cells and tissues compared to the controls [37, 38]. Oncogenic effects of miR-155 were suggested to be due to downregulation of a tumor suppressor CDC73 in OSCC [39]. Similarly, miR-27a was shown to downregulate expression of and to inhibit tumor suppressor function of microcephalin 1 (MCPH1) in OSCC cells [40].

## 4. miRNAs as Tumor Suppressors in OSCC

Several miRNAs have been shown to be downregulated in OSCC. Accordingly, functional studies have demonstrated tumor suppressive roles for these miRNAs in OSCC tumorigenesis. miR-320 was downregulated in OSCC-derived cell-lines and tissue specimens, with its expression correlating inversely with the vascularity. Hypoxia suppressed miR-320 expression through HIF-1*α* and increased the expression of neuropilin 1 (NRP1) and promoted the motility and tube formation ability of endothelial cells via vascular endothelial growth factor (VEGF) signaling pathway, resulting in tumor angiogenesis [41].

The function of miR-7 has been characterized as a tumor suppressor in several human cancers, including glioblastoma, breast cancer, and OSCC among others. A number of protooncogenes were experimentally confirmed as its target genes, including insulin receptor substrate 1 (IRS1), insulin receptor substrate 2 (IRS2), epidermal growth factor receptor (EGFR), v-raf-1 murine leukaemia viral oncogene homologue 1 (RAF1), and p21/CDC42/RAC1-activated kinase 1 (PAK1) [42–44]. Jiang et al. showed that miR-7 regulated IGF1R/IRS/PI3K/Akt signaling pathway by posttranscriptional regulation of insulin-like growth factor 1 receptor (IGF1R) in cells derived from tongue squamous cell carcinoma (TSCC, the most common subtype of OSCC) cells [45]. Similarly, studies have demonstrated that IGF1R and mammalian target of rapamycin (mTOR), components of IGF1R signaling pathway, are target genes of another tumor suppressor miRNA, the miR-99a [46, 47]. Downregulation of miR-99a was observed in OSCC patient specimens and cell-lines [46, 47], especially in OSCC patients with lymphovascular invasion [46], suggesting a role for miR-99a in lymphovascular invasion. In addition, miR-99a induced apoptosis and inhibited OSCC cell proliferation, migration, and invasion* in vitro* as well as lung colonization* in vivo* [46, 47].

miR-218 has been shown to be epigenetically (DNA hypermethylation) silenced in OSCC tissue specimens and to have a tumor suppressive function by regulating the expression of rapamycin-insensitive component of mTOR, Rictor [48]. DNA hypermethylation has been suggested as one of the mechanisms for the downregulation of miR-9 in OSCC and oropharyngeal carcinoma [49]. Lentivirus-mediated miR-9 overexpression in highly aggressive tumor cells led to significant inhibition of proliferation* in vitro* and* in vivo*. These tumor suppressive functions were suggested to be mediated via targeting CXC chemokine receptor 4 (CXCR4) gene and Wnt/*β*-catenin signaling pathway [50].

Accumulating evidence suggests a critical role for EMT in tumor progression, invasion, and metastasis and acquisition of stem-like phenotype [51]. Findings from a number of studies point towards a role of miRNAs in the regulation of EMT and EMT-related malignant phenotypes in OSCC cells. Different studies have shown a role for miR-138 in the suppression of EMT, cell proliferation, migration, and invasion in HNSCC-derived cells. At the molecular level, miR-138 regulated the expression of key EMT-related molecules like Fos-like antigen 1 (FOSL1), vimentin (VIM), zinc finger E-box-binding homeobox 2 (ZEB2), enhancer of zeste homologue 2 (EZH2), RhoC, and ROCK2 [52–54]. Furthermore, miR-138 was suggested to suppress the expression of prometastatic RhoC and other downstream signaling molecules FAK, Src, and Erk1/2 in HNSCC-derived cells [55]. Likewise, miR-181a was shown to inhibit Twist1 mediated EMT, metastatic potential and cisplatin induced chemoresistance in TSCC cells [56].

Recent studies have shown that miRNAs play a crucial role in the regulation of extracellular matrix (ECM) components, such as matrix metalloproteinases (MMPs) and integrins. Lu and coworkers reported that miR-29a was underexpressed in OSCC tissues and inhibited the expression of MMP2 by directly binding to the MMP2 3′-UTR. Functionally, miR-29a inhibited invasion and antiapoptosis of OSCC-derived cells [57]. Further functional studies revealed that transfection with miRNA-29a mimics attenuated invasive potential, increased apoptosis rate, and enhanced chemosensitivity of OSCC cell-lines to cis-platinum (CDDP) [57]. miR-124 was found to be downregulated in OSCC and its forced expression suppressed OSCC cell migration and invasion through downregulation of ITGB1 expression [58]. Furthermore, miR-491-5p was shown to suppress invasion and metastatic potential of OSCC cells* in vitro* and* in vivo* by targeting the expression of G-protein-coupled receptor kinase-interacting protein 1 (GIT1), which further regulated the expression of focal adhesions, steady-state levels of paxillin, phospho-paxillin, phospho-FAK, EGF/EGFR-mediated extracellular signal-regulated kinase (ERK1/2) activation, and MMP2/9 levels and activities [59].

A miRNA cluster, miR-17-92, including miR-17, miR-19b, miR-20a, and miR-92a, was found to be significantly downregulated in a more migratory OSCC-derived TW2.6 MS-10 cells as compared to the less migratory TW2.6 cells. Overexpression of this cluster was found to decrease the migratory ability of OSCC cell-lines. Through a bioinformatics screening analysis and 3′-UTR reporter assay, integrin (ITG) *β*8 was identified to be a direct target of miR-17/20a in OSCC cells [60]. Likewise, miR-375 was shown to be downregulated in HNSCC and to function as a tumor suppressor by regulating the expression of AEG-1/MTDH, CIP2A (cancerous inhibitor of protein phosphatase 2A). Transient transfection of miR-375 in HNSCC-derived cells reduced the expression of CIP2A (cancerous inhibitor of protein phosphatase 2A) [61, 62]. Furthermore, miR375 sensitized TNF-*α*-induced apoptosis probably through inhibiting NF-*κ*B activation* in vitro* [63]. Previous studies have suggested miR-34a, which was frequently downregulated in a number of tumor types, to function as a tumor suppressor. Ectopic expression of miR-34a suppressed proliferation and colony formation of HNSCC cells by downregulation of E2F transcription factor 3 (E2F3) and survivin in the* in vitro* and* in vivo* models [64]. miR-34a further led to the inhibition of tumor angiogenesis by blocking VEGF production as well as by directly inhibiting endothelial cell functions [64]. miR-125b, another downregulated miRNA in OSCC, was able to inhibit proliferation rate and to enhance radiosensitivity to X-ray irradiation via downregulation of ICAM2 mRNA expression in OSCC-derived cells [65]. Likewise, miR-145 was found to be frequently downregulated in OSCCs [66] and to inhibit OSCC cell proliferation and colony formation [67].

## 5. Diagnostic and Prognostic Value of miRNAs in OSCC

Distinct expression profile of miRNA in OSCC and oral prelalignant tissue specimens compared to the normal controls offers the use of specific miRNA(s) signature for early stage diagnosis and prediction of OSCC prognosis [16, 17]. In addition, miRNAs possess the following unique properties which make them attractive diagnostic and prognostic tool in OSCC. Firstly, they are abundantly expressed in OSCC and control tissues and hence their isolation and quantification are convenient and reproducible. Secondly, several OSCC-related miRNAs are secreted in bodily fluids such as serum, plasma, and saliva [68] making them very useful for noninvasive clinical application. Candidate miRNAs reported to be relevant for OSCC diagnostics and prognosis are summarized in Table 2.

### 5.1. miRNA as Diagnostic Biomarkers

The use of a specific miRNA signature as a diagnostic tool in OSCC has been suggested by a number of recent studies. miR-16 and let-7b were highly upregulated in sera from patients with OSCC and oral carcinoma* in situ*, while miR-338-3p, miR-223, and miR-29a were highly downregulated as compared to the matched controls. ROC analysis indicated that the signature of five miRNAs (miR-16, let-7b, miR-338-3p, miR-223, and miR-29a) might be useful as a biomarker for oral cancer detection (AUC > 0.8) [69]. Lin et al. showed that the plasma levels of miR-24 in OSCC patients were significantly higher than in the control individuals [70]. Likewise, the elevated plasma levels of miR-21 and miR-146a were suggested to have a diagnostic value in OSCC [33, 71]. Expression level of miR-31 in saliva was found to be significantly increased in patients with OSCC of all clinical stages as compared to that of the healthy controls. The high salivary level was significantly reduced after excision of OSCC lesion, indicating that the main contributor for miR-31 upregulation was OSCC lesion [72]. In addition, increased expression of miR-27b in saliva of OSCC patients was suggested as a valuable biomarker to identify OSCC patients by ROC curve analyses [73]. However, another study showed a downregulated expression of miR-27b in both the tumor tissues and the plasma of OSCC patients [74]. Further research is therefore required to validate the above findings and elucidate the molecular mechanism of different levels of miR-27b in saliva and plasma in OSCC.

In addition to their potential use in OSCC diagnosis, several miRNAs were suggested to be important in the earlier diagnosis and prediction of malignant transformation of oral premalignant lesions/conditions. Dang et al. showed a significantly higher methylation frequency of miR-137 promotor in patients with oral lichen planus (35%) and OSCC (58.3%) as compared to the absence of methylation in normal controls, suggesting that the methylation status of miR-137 might be a valuable biomarker in the prediction of malignant transformation of OLP [75]. In saliva, significantly different expressions of miR-10b, miR-145, miR-99b, miR-708, and miR-181c were observed in progressive low grade dysplasia (LGD) as compared to nonprogressive LGD leukoplakia patients [76].

### 5.2. miRNA as Prognostic Biomarkers

The expression patterns of certain miRNAs have been found to correlate with clinical stage, lymph node metastasis, and patient survival, indicating that these miRNAs can act as prognostic predictors in OSCC. Higher expression levels of miR-21 in TSCC correlated with advanced clinical stage, poor differentiation, and lymph node metastasis [16]. Moreover, multivariate analysis showed that expression level of miR-21 could be used as an independent prognostic factor for TSCC patients' survival [16]. Similarly, prognostic value of miR-21 in OSCC/HNSCC was reported in another study [26]. miR-31, miR-17/20a, miR-125b, miR-155, miR-181, miR-375 and miR-491-5p, miR-205, and miR-let7d were found to be associated with lymph node metastasis and poor OSCC patient survival [38, 59, 60, 65, 72, 77–80].

### 5.3. miRNA as Target for OSCC Therapy

The ability to manipulate miRNAs expression and function by local and systemic delivery of miRNA inhibitors (anti-miRNA oligonucleotides or miRNA sponges [81, 82]) or miRNA mimics [82] has recently gained immense interest as novel therapeutic approach. This treatment approach came into light after the first successful anti-miRNA oligonucleotides based human clinical trial in 2011 for the treatment of hepatitis C virus infection (reviewed in [83]). Recent identification of key miRNAs with either oncogenic or tumor suppressive functions in OSCC has opened up new possibilities for miRNA based OSCC therapy. The advantage of miRNA based cancer therapy lies in the ability of miRNAs to concurrently target multiple effectors of pathways involved in cell proliferation, differentiation, and survival [82]. Accordingly, several* in vitro* and* in vivo* studies, employing strategies to suppress the function of oncogenic miRNA and/or restore the tumor suppressive miRNAs, have reported significant inhibition of aggressive OSCC phenotypes. For example, inhibiting miR-21 by anti-miRNA oligonucleotides has been shown to inhibit survival, anchorage-independent growth [16], and invasion [18] of OSCC cells. Likewise, restoration of miR-99a level by miR mimic transfection markedly suppressed proliferation and induced apoptosis of TSCC cells [47].

Resistance to chemotherapy and resistance to radiotherapy are major challenges in the management of OSCC patients as significantly high proportions of OSCC lesions fail to respond to these treatment modalities. Recent studies have linked resistance to chemotherapy and radiotherapy in OSCC to altered miRNA expression and function. Dai et al. have correlated a miRNA signature (downregulation of miR-100, miR-130a, and miR-197 and upregulation of miR-181b, miR-181d, miR-101, and miR-195) in HNSCC cells with multiple drug resistance phenotypes* in vitro* [84]. In another study, low expression of miR-200b and miR-15b in TSCC was associated with chemotherapeutic resistance and poor patient prognosis [85]. Similarly, higher expression of miR-196a was reported to be associated with recurrent disease and resistance to radiotherapy in HNSCC [86]. The miRNA signature(s) related to therapeutic resistance has also been used experimentally to revert the resistance phenotypes. For example, inhibition of miR-21 by anti-miRNA oligonucleotides has been shown to inhibit chemoresistance in OSCC cells [24, 87]. Likewise, forced expression of miR-125b has been reported to enhance radiosensitivity in OSCC cells [65]. Recently, nanoparticle based delivery of miRNAs was suggested as a promising approach in the treatment of HNSCC [88]. Despite these promising results, more in-depth studies are necessary to better understand the effective delivery system for optimal uptake and to minimize degradation of miRNA based drugs in the* in vivo* situation.

## 6. Conclusions

Alteration in the expression pattern of miRNA is a common finding in OSCC tumorigenesis. Several altered miRNAs seem to play critical roles in the initiation and progression of OSCC by functioning either as oncogenes or as tumor suppressors. Specific miRNA signatures identified from tumor specimens, serum/plasma, or saliva from OSCC patients have a potential to be clinically useful in the diagnosis, prognosis, and therapeutic targets in OSCC. Nevertheless, it will be a big challenge ahead to translate these promising findings to clinic before the following issues will be fully addressed. Firstly, findings from several studies are based on limited number of patient materials from different sublocations of oral cavity, which lead to more heterogeneous data and reduced statistical power. Additionally, use of different expression profiling platforms (such as microarray or PCR) with different normalizing strategies leads to inconsistent miRNA expression results. Hence, a comprehensive miRNA profiling including larger number of paired tissue specimens of oral premalignant lesions/conditions, primary OSCC, and metastasis will enable us to identify miRNAs involved in stepwise tumorigenesis and metastatic process of OSCC. The identified miRNAs will pave the way for their future clinical use in the diagnosis, prognosis and therapy of OSCC.

## Figures and Tables

**Table 1:** Summary of miRNAs and associated signal pathways/target genes in OSCC/HNSCC.

miRNA	Up/downregulation	Target genes/associated pathways	Ref.
miR-21	Up	PDCD4	[23]
		TPMI	[16]
		RECK	[26]
		CLU	[22]
		DKK2-Wnt/*β*-catenin	[18]
		Smad7-TGF*β*1	[27]
		HA/CD44-Nanog/Stat3-PDCD4, IAPs	[24]

miR-31	Up	FIH-HIF-EVGF	[28]

miR-31^*∗*^	Up	FGF3	[29]
		RhoA	[32]

miR-134	Up	WWOX	[36]

miR-146a	Up	IRK1, TRAF6, and NUMB	[33]

miR-155	Up	CDC73	[39]

miR-7	Up	RECK	[26]
		IGF1R-Akt	[45]

miR-9	Down	CXCR4-Wnt/*β*-catenin	[50]

miR-17/20a	Down	ITG*β*8	[60]

miR-29a	Down	MMP2	[57]

miR-34	Down	E2F3, survivin, and VEGF	[64]
		SIRT6	[89]

miR-99a	Down	IGF1R	[46]

miR-124	Down	ITGB1	[58]

miR-125b	Down	ICAM2	[65]

miR-138	Down	FOSL1	[52]
		VIM, ZEB2, EZH2	[53]
		RhoC, and RoCK2	[54]

miR-140-5p	Down	ADAM10, ERBB4, PAX6, and LAMC1	[90]

miR-145	Down	c-Myc, Cdk6	[67]

miR-181a	Down	K-ras	[91]
		Twist1	[56]

miR-205	Down	IL-24, caspase-3/-7,	[92]
		and Axin-2	[93]

miR-218	Down	mTOR-Rictor-Akt	[48]

miR-320	Down	HIF-1*α*-NRP1-VEGF	[41]

miR-357	Down	CIP2A-MYC	[61]
		AEG-1/MTDH	[62]

miR-419-5p	Down	GIT1	[59]
		EGFR-ERK1/2-MMP2/9	

miR-483-3p	—	API5, BRIC5, and RAN	[94]

miR-196a	Up	MAMDC2	[95]

miR-26a/b	Down	TMEM184B	[96]

**Table 2:** miRNA deregulation and relevance to OSCC diagnosis and prognosis.

miRNA(s)	Source	Up/downregulation (OSCC versus normal control)	Diagnostic/prognostic relevance	Ref.
miR-16, Let-7b	Serum	Up	Yes/ND	[69]^a^
miR-223, miR-29a,	Serum	Down		
and miR-338-3p				

miR-24	Plasma	Up	Yes/ND	[70]

miR-146a	Tissue/plasma	Up	Yes/ND	[33]

miR-21	Tissue	Up	Yes/ND	[71]^b^
	Plasma	Up	Yes/yes	
	Tissue	Up	ND/yes	[16]^c^
	Tissue	Up	ND/yes	[26]
	Tissue	Up	Yes/yes	[17, 80]^b^
	Tissue	Up	ND/yes	[20]^d^

miR-31	Saliva	Up	Yes/ND	[72]

miR-27b	Saliva	Up	Yes/ND	[73]

miR-125b	Tissue	Down	ND/yes	[65]

miR-491-5p	Tissue	Down	ND/yes	[59]

miR-181	Plasma/tissue	Up	Yes/yes	[77]

miR-375	Tissue	Down	ND/yes	[78]^b^

miR-205 and Let-7d	Tissue	Down	ND/yes	[79]^b^

miR-155	Tissue	Up	ND/yes	[38]
	Tissue	Up	Yes/yes	[37]

miR-21-3p	Tissue	Up	ND/yes	[97]
miR-141-3p				
miR-96-5p				
miR-130b-3p				

miR-196a/b	Tissue	Up	Yes/yes	[98]
miR-196a	Plasma	Up	Yes/yes	

miR-211	Tissue	Down	ND/yes	[99]

^a^OSCC group also consists of lesions with carcinoma *in situ*; ^b^HNSCC specimens; ^c^TSCC; ^d^expression examined in the tumor stroma; Ref.: references; ND: not determined.
